# *Barsine podbolotskayae* sp. n. from Flores Island, Lesser Sunda Archipelago, Indonesia (Lepidoptera, Erebidae, Arctiinae)

**DOI:** 10.3897/zookeys.768.24345

**Published:** 2018-06-19

**Authors:** Vitaly M. Spitsyn, Ivan N. Bolotov

**Affiliations:** 1 Northern Arctic Federal University, Severnoy Dviny Emb. 17, 163002, Arkhangelsk, Russia; 2 Federal Center for Integrated Arctic Research of the Russian Academy of Sciences, Northern Dvina Emb. 23, 163000, Arkhangelsk, Russia

**Keywords:** East Nusa Tenggara, island biogeography, lichen moths, Wallacea

## Abstract

Herein *Barsine podbolotskayae* **sp. n.** (Lepidoptera: Erebidae: Arctiinae) is described from Flores Island, Lesser Sunda Archipelago, Indonesia. This local endemic species externally resembles *Barsine exclusa* Butler, 1877 from Sundaland and the Andaman Islands but differs by marking patterns and male genitalia structure.

## Introduction

The Lesser Sunda Archipelago is a vast island group, which includes several large islands such as Timor, Sumbawa, Sumba, and Flores. This archipelago together with Sulawesi and the Moluccas is a part of the Wallacea. This region consists of two mostly distinct transition zones between the Oriental and Australasian biotas, i.e., a humid forest northerly zone from the Philippines to Sulawesi and the Moluccas, and a seasonal forest and savannah southerly zone along the Lesser Sunda chain from Java to Timor (Holloway and Jardine 1968). Contacts between the two zones has mostly been through Sulawesi, whose fauna is mainly Oriental, whereas the Moluccas are predominantly Australasian (Holloway 2003). The major discontinuity between the Oriental and Australasian Lepidoptera is Weber’s Line of Faunal Balance that runs between Sulawesi and the Moluccas and then south to east of Timor (Holloway and Jardine 1968). There is a continuum of species turnover from Oriental to Australasian affinity from Java to Timor, but there is also some endemism within the Lesser Sunda Islands (Holloway 2003; Lohman et al. 2011; Bolotov et al. 2017, 2018; Spitsyn et al. 2018).

The lichen moth genus *Barsine* Walker, 1854 (Lepidoptera: Erebidae: Arctiinae) (type species: *Barsine defecta* Walker, 1854) is widespread across the Oriental tropics from the mainland to the Lesser Sunda Islands and Moluccas, but it is replaced by *Cyme* Felder, 1861, another morphologically similar and possibly related genus, in New Guinea and Australia (Holloway 2001). To the best of our knowledge, published occurrences of any species of *Barsine* from Flores are lacking. As for the entire Lesser Sunda Islands, *B. dohertyi* (Rothschild, 1913) is the only known member of this group that has been described from Sumbawa (Rothschild 1913; Holloway 1982, 2001). Additionally, *B. sanguitincta* (Hampson, 1900) is the only known species of the genus from the Moluccas (Hampson 1900; Holloway 1982, 2001).

The present short correspondence describes *Barsine podbolotskayae*, a species new to science that occurs in Flores.

## Materials and methods

This study is based on the materials from the collection of the Russian Museum of Biodiversity Hotspots (RMBH thereafter) of the Federal Center for Integrated Arctic Research of the Russian Academy of Sciences, Arkhangelsk, Russia. The genitalia were dissected and mounted on a glass slide with Histofluid® (Paul Marienfeld GmbH & Co., Germany). The images of specimens were taken with a Canon EOS 650D camera (Canon, Tokyo, Japan). The photos of the genitalia were obtained using two research stereomicroscopes (SteREO Discovery.V8 and AXIO Zoom.V16, Carl Zeiss, Germany).

## Results

### Barsine podbolotskayae sp. n.

Taxon classificationAnimaliaLepidopteraErebidae

http://zoobank.org/43B36AA3-CA81-4AA8-A163-FD55288A6743

Figs 1 , 2 , 3

#### Type material.

Holotype: ♂, INDONESIA, Lesser Sundas, East Nusa Tenggara, Flores Island: Sano Ngoang Lake, camp site, secondary mountain forest with old nutmeg trees on a hill slope, 8°42'33.50"S, 119°59'51"E, 21–22 January 2015, Bolotov leg., in RMBH (voucher no. Sph0682). Paratypes: 3♂♂, 2♀♀, same data as holotype, all in RMBH (vouchers nos. Sph0683, Sph0731, Sph0732, Sph0733 and Sph0734).

#### Diagnosis.

The new species externally resembles *Barsine exclusa* Butler, 1877, similarly patterned on forewing, both showing the outer boundary of the discal patch of ground color lined by W-shaped postmedial line. However, the new species differs by the lack of a discal spot within that patch (vs presence of a gray discal spot), its bright crimson-colored hindwing (vs pinkish-yellow or pinkish-white), and stronger developed gray markings on forewing, with broad dark shading beyond the postmedial line (vs weaker developed gray markings and lack of broad dark terminal shading). The male of *B. podbolotskayae* sp. n. can be distinguished from those of all other known species of *Barsine*, including *B. exclusa*, by a dorsally directed, robust, spine-like central costal process of the valve and a bundle of dorsally directed, long setae proximal to it (vs lack of such features). It differs from *B. exclusa* also by the narrower neck of cucullus, this rounded and apically setose (vs wider neck of acutely pointed cucullus).

#### Description.

**Figure 1.**
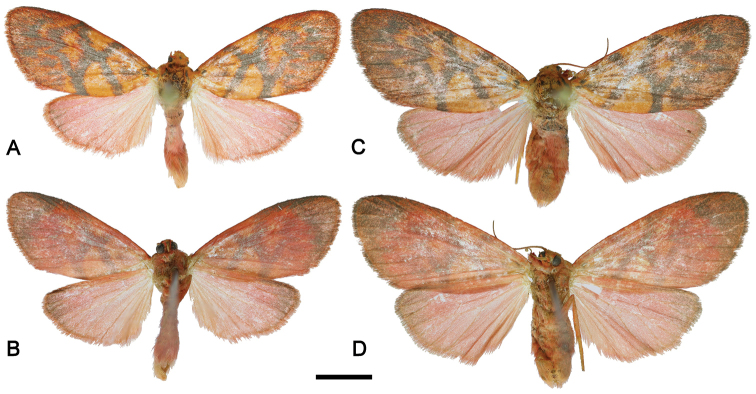
Type specimens of *Barsine podbolotskayae* sp. n., Flores Island, Indonesia. **A** Holotype male (upperside) **B** Holotype male (underside) **C** Paratype female (upperside) **D** Paratype female (underside). Scale bar 5 mm (photographs Vitaly M. Spitsyn).

**Male.** Wingspan 22–24 mm, forewing length 11–12 mm (N = 4). Eye black; antenna red dorsally and gray ventrally; frons red-orange, vertex orange with black spot in the middle; labial palpus stout, straight and short (equal to eye diameter), brick red. Thorax dorsally orange-red; patagium and tegula brick-red with black spot in the middle; underside and legs crimson. Forewing upperside brick red, with a few indistinct gray dots in basal area, angled antemedial and medial wide gray lines joined at middle in shape of ‘X’ mark (Fig. 1A). Outer boundary of discal patch of ground color W-shaped due to inwardly projected jags from postmedial line, this gray too; distal field with extended dark gray suffusion and some indistinct gray dots. Hindwing upperside uniformly bright crimson. Underside of both wings crimson-red, with brownish shading near apex (Fig. 1B). Abdomen light crimson.

**Female.** Wingspan 27–28 mm, forewing length 13–14 mm (N = 2). Patterned as in male (Fig. 1C–D).

**Figure 2.**
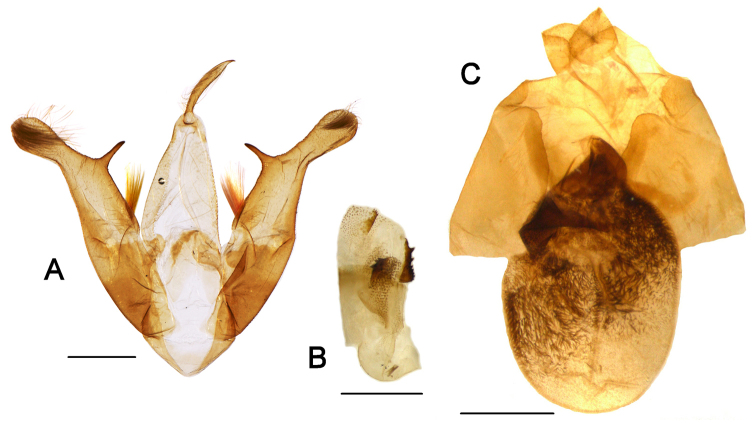
Male and female genitalia of *Barsine podbolotskayae* sp. n., Flores Island, Indonesia. **A** Male genitalia (holotype) **B** Aedeagus (holotype) **C** Female genitalia (paratype). Scale bar 1 mm (photographs Vitaly M. Spitsyn).

**Male genitalia.** Tegumen comparatively long and broad; saccus broad, short, V-shaped (Fig. 2A). Valva narrow, elongated; costa with single robust, straight spine-like central process, approximately perpendicular, and subbasal bundle of stiff, dorsally directed long setae; neck of cucullus narrower than cucullus, this membranous, rounded and distally setose; sacculus weakly developed, consisting of a fold on the inner surface of the valva. Uncus long and thin, laterally compressed, broadened subapically and slightly curved, with spine-like apex. Scaphium narrow. Juxta broad, U-shaped, weakly sclerotized. Aedeagus short and broad; vesica broad, with two sclerotised plates bearing numerous teeth, and two granulose fields (Fig. 2B).

**Female genitalia.** Ostium bursae broad, funnel-shaped, sclerotized, fused with the seventh sternite; antrum not traced; ductus bursae very short, sclerotized (Fig. 2C). Bursa copulatrix elliptical, thickly covered with long spinules in medial and posterior sections, with strongly sclerotized cervix. Apophyses anteriores and posteriores of similar length, long and thin. Papillae anales (ovipositor lobes) broad, with rounded edges, covered densely with long fine setae.

#### Etymology.

This new species is named in memory of Dr. Marina V. Podbolotskaya (1956–2014), a well-known Russian entomologist.

#### Distribution.

**Figure 3.**
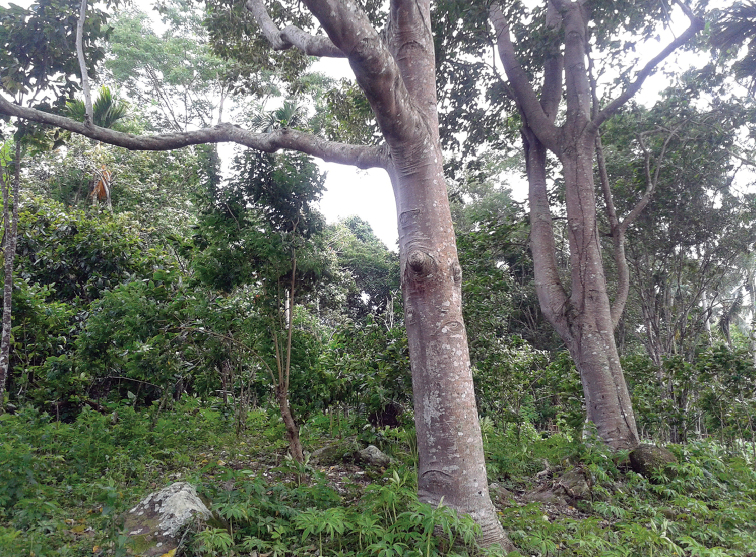
Type locality of *Barsine podbolotskayae* sp. n.: Flores Island, shore of Lake Sano Nggoang, secondary mountain forest with old nutmeg trees on a hill slope (photographs M. Albarran Valle).

Flores Island, Lesser Sunda Archipelago; only known from the type locality (Fig. 3).

#### Conservation status.

The new species appears to be Critically Endangered (CR B1a) because it is known to exist at only a single location.

#### Remarks.

Here we placed *B. podbolotskayae* sp. n. within the genus *Barsine* but its placement is in need of further investigation. Features of the costal margin of valva have so far not been found in any other known members of the genus (cf. Černý and Pinratana 2009; Holloway 2001; Bucsek 2012; Volynkin and Černý 2017a, b, c, 2018), so that solely on these grounds *B. podbolotskayae* sp. n. might represent another genus. Nonetheless, we hesitate to erect a new genus for this species pending upon a thorough review of *Barsine*, *Cyme* and other closely related genera, whose systematic relationships are still largely unclear and need to be phylogenetically assessed (Holloway 2001; Volynkin and Černý 2017c).

## Discussion

The fauna of the Lesser Sunda Islands comprises two species of *Barsine*: *B. podbolotskayae* sp. n. (Flores) and *B. dohertyi* (Sumbawa). Holloway (2001) noted that that the *Barsine* is a genus with clear affinities to the mainland Southeast Asia and Sundaland, the species richness of which decreases abruptly east of the Wallace Line. Mainland Southeast Asia is considered the most probable evolutionary hotspot of this group (Holloway 2001; Černý and Pinratana 2009; Bucsek 2012). Possible sister relationships between *Barsine* and *Cyme* (Holloway 2001) are in need of future research using a molecular approach as this may uncover putative ancient connections between the Oriental and Australasian faunas (Holloway 2003).

## Supplementary Material

XML Treatment for Barsine podbolotskayae
